# A High-Density 4H-SiC MOSFET Based on a Buried Field Limiting Ring with Low Q_gd_ and R_on_

**DOI:** 10.3390/mi16040447

**Published:** 2025-04-10

**Authors:** Wenrong Cui, Jianbin Guo, Hang Xu, David Wei Zhang

**Affiliations:** School of Microelectronics, Fudan University, Shanghai 200433, China

**Keywords:** SiC, high density, gate oxide protection, distributed grounding

## Abstract

In this study, we propose an optimized shield gate trench 4H-SiC structure with effective gate oxide protection. The proposed device has a split trench with a P+ shield region, and the P+ shield is grounded by the middle deep trench. Our simulation results show that the peak electric field near the gate oxide is almost completely suppressed. Compared with a conventional P+ shield device, our proposed structure achieves a 78% reduction in the Q_gd_ and a 108% increase in the FoM (figure of merit) simultaneously. Additionally, it is estimated that the device cell pitch can be reduced to 1.8 μm with a R_on_ below 0.94 mΩ·cm^2^, in theory. These demonstrated device performance metrics, as well as its simple structure and good compatibility, make our proposed SiC MOSFET highly attractive for future high-performance applications.

## 1. Introduction

Compared with conventional silicon (Si) material, silicon carbide (SiC) exhibits three fundamental material advantages that are critical for power electronics applications: (1) a wide bandgap (3.26 eV for 4H-SiC vs. 1.12 eV for Si), enabling high-temperature operation up to 600 °C; (2) an exceptionally high critical breakdown field strength (2.8 MV/cm for SiC versus 0.3 MV/cm for Si), allowing thinner drift layers and higher voltage blocking capability; and (3) superior thermal conductivity (4.9 W/cm·K for SiC compared to 1.5 W/cm·K for Si), which significantly improves heat dissipation [1,2].

From a fabrication perspective, the design methodology and processing techniques for SiC metal oxide semiconductor field-effect transistor (MOSFET) devices maintain strong compatibility with established silicon-based MOS device manufacturing flows. This includes similar photolithography processes, oxide deposition techniques, and metallization schemes, thereby facilitating the adoption of SiC MOSFETs as direct replacements for silicon power MOSFETs in existing power electronic systems [3,4].

The current technological roadmap for SiC MOSFET development prioritizes four key performance metrics: (i) the minimization of cell dimensions (targeting sub-micron features to increase the current density); (ii) a reduction in specific on-resistance (R_on,sp_) through optimized doping profiles and channel mobility enhancement; (iii) the lowering of switching losses (E_off_) via improved minority carrier lifetime control; and (iv) the enhancement of the gate oxide reliability through advanced interfacial passivation techniques [5,6]. Thermal management considerations are becoming increasingly crucial as device densities increase.

In terms of the device architecture, contemporary commercial SiC power MOSFETs predominantly employ either planar or trench gate configurations [7]. The planar topology, while offering simpler fabrication and better gate oxide integrity, presents inherent geometrical constraints in minimizing the cell pitch due to the lateral separation required between adjacent JFET regions. In contrast, trench architectures enable more aggressive pitch scaling through vertical channel formation.

A reduction in the cell pitch yields multiple performance benefits: (a) a substantial decrease in the die area (enabling higher current ratings per unit chip size); (b) a lower specific on-resistance (R_on,sp_) through increased channel density; (c) reduced input and output capacitances (C_iss_ and C_oss_) due to smaller electrode overlaps; and (d) markedly improved switching efficiency, as evidenced by reduced energy losses during both turn-on (E_on_) and turn-off (E_off_) transitions [8,9,10]. Recent studies have demonstrated that every 1 μm reduction in cell pitch can provide an approximate 15–20% improvement in the overall figure of merit (FoM = R_on,sp_ × E_off_).

Nonetheless, trench SiC MOSFETs encounter certain issues, primarily associated with the gate oxide. Particularly, there exists a strong electric field at the trench bottom, which may give rise to gate oxide premature breakdown [11,12,13]. Numerous approaches have been proposed to mitigate the electric field based on numerical simulations [14,15,16,17,18,19,20,21,22]; however, these methods often entail complex structures and pose challenges in terms of process feasibility.

In contemporary SiC MOSFET designs, the self-aligned additional P+ shielding layer is commonly employed to significantly decrease the electric field. However, this approach also leads to an increase in the device’s R_on_, and the presence of a floating P+ region also introduces the issue of the charge storage effect [23,24].

A grounding P+-shield region at the bottom of a trench offers significant benefits, especially in terms of improved gate oxide protection, a well-researched area. However, it is difficult to achieve P+ grounding at the bottom of the trench in both process and device structure, which is currently a research focus of research institutions including Infineon and Rohm. Infineon has addressed this challenge by connecting the shield P+ to a 0 potential through a semi-enclosed trench structure [25]. Nevertheless, this technique results in the wastage of half of the channel area, thereby limiting further advancements in chip density. Rohm adopts a grounded source trench on both sides of each gate trench, which also faces the problem of low integration.

To address these challenges, we present a novel solution in this study: a buried field limiting ring device (P+ shield) grounded through a deep trench. The trench gate structure is divided into three distinct sections and the two side sections serve as conventional gates. Our proposed device fabrication process ensures full compatibility with high-density integration, high breakdown voltage (BV), low R_on_, and various other advantages.

## 2. Structure Design and Fabrication Process

**Figure 1:**
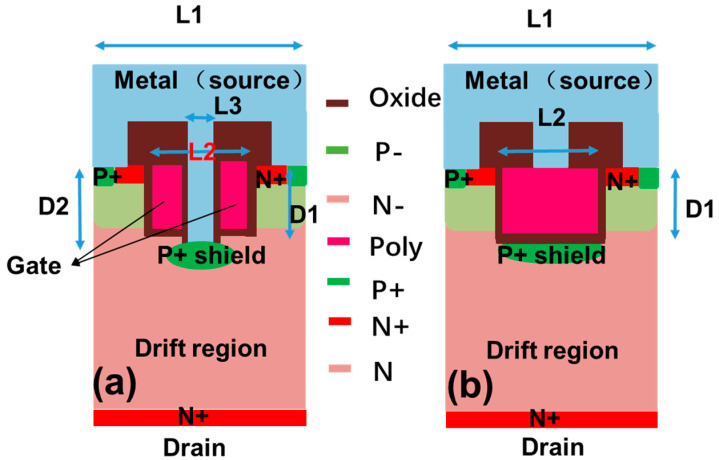
Schematic structure of (**a**) the proposed SiC MOS and (**b**) conventional P+ shield trench SiC MOS.

Figure 1a,b depict the schematic structures of the proposed and conventional P+ shield 4H-SiC trench gate MOSFETs. And the main structure and process parameters of the device are shown in Table 1. In the proposed device, the trench gate is divided into symmetrical components through the incorporation of a deep trench structure. The deep trench is embedded into the drift region to form a P+ shield grounded channel. As a result, the inverted p-n junction in the OFF state is pushed down into the drift region, effectively confining the depletion region outside the channel region. As a result, the peak electric field near the gate oxide is almost completely suppressed. In comparison to the conventional device, the proposed device maintains identical structures and doping profiles. The 4H-SiC substrate possesses a doping concentration of 7.0 × 10^15^ cm^−3^ to obtain a BV higher than 1200 V. The normal trench gate depth (1 μm), cell pitch (2.4 μm), total trench width (1.6 μm), and ion implantation conditions for the P+ shield region are also kept consistent. The doping concentration of the P+ shield and P base are 2 × 10^19^ cm^−3^ and 2.5 × 10^17^ cm^−3^, respectively.

**Figure 2:**
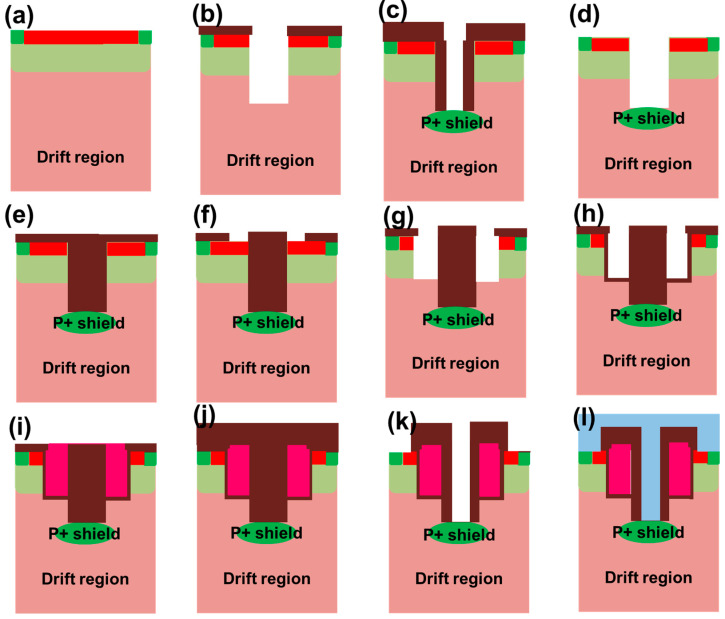
One possible fabrication process for the proposed SiC MOSFET. (**a**) The N-drift is formed on the N+ substrate using epitaxial techniques, and then, the emitter P+N+ is formed by photolithography (PEP) and ion implantation (ion); (**b**) oxide deposition, PEP, oxide etching, and SiC etching to form a deep trench; (**c**) oxide deposition, etching to form side walls, and self-aligned bottom P-well injection; (**d**) annealing for ion activation and oxide etching; (**e**) oxide deposition and chemical mechanical polishing (CMP); (**f**) PEP and oxide etching; (**g**) SiC etching; (**h**) thermal oxidation process to form the gate oxide; (**i**) polysilicon deposition and etching; (**j**) oxide deposition; (**k**) PEP and oxide coating etching to form ground channels in the bottom of deep trench; (**l**) metal deposition.

Figure 2 illustrates one available fabrication process for the proposed SiC MOSFET. (a) The N-drift is formed on the N+ substrate using epitaxial techniques, and then, the emitter P+N+ is formed by photolithography (PEP) and ion implantation (ion); (b) oxide deposition, PEP, oxide etching, and SiC etching to form a deep trench; (c) oxide deposition, etching to form side walls, and self-aligned bottom P-well injection; (d) annealing for ion activation and oxide etching; (e) oxide deposition and chemical mechanical polishing (CMP); (f) PEP and oxide etching; (g) SiC etching; (h) thermal oxidation process to form the gate oxide; (i) polysilicon deposition and etching; (j) oxide deposition; (k) PEP and oxide coating etching to form ground channels in the bottom of deep trench; (l) metal deposition.

All of the process steps are compatible with current manufacturing processes and require only one additional lithography mask. The proposed device has advantages of a simple structure and low cost, as the manufacturing cost is mainly related to the duration of lithography.

## 3. Simulation Results and Discussion

Two-dimensional (2D) simulation models are developed, utilizing the existing SiC MOSFET process as the foundation. The proposed device and conventional devices with and without a P+ shield are chosen to be compared. The crucial models are taken into account, including Shockley–Read–Hall recombination, Auger recombination, Okuto–Crowell impact ionization, incomplete dopant ionization, doping-dependent transport, band narrowing, barrier lowering, anisotropic material properties, non-local tunneling, and so on. The effect of the SiO_2_/SiC interface traps is taken into consideration and the charge concentration is 2 × 10^11^ cm^−2^. And the channel mobility below the SiC/SiO_2_ interface is about 20–50 cm^2^·V^−1^s^−1^ by modifying the parameters of the Lombardi model.

A. Breakdown characteristics

**Figure 3:**
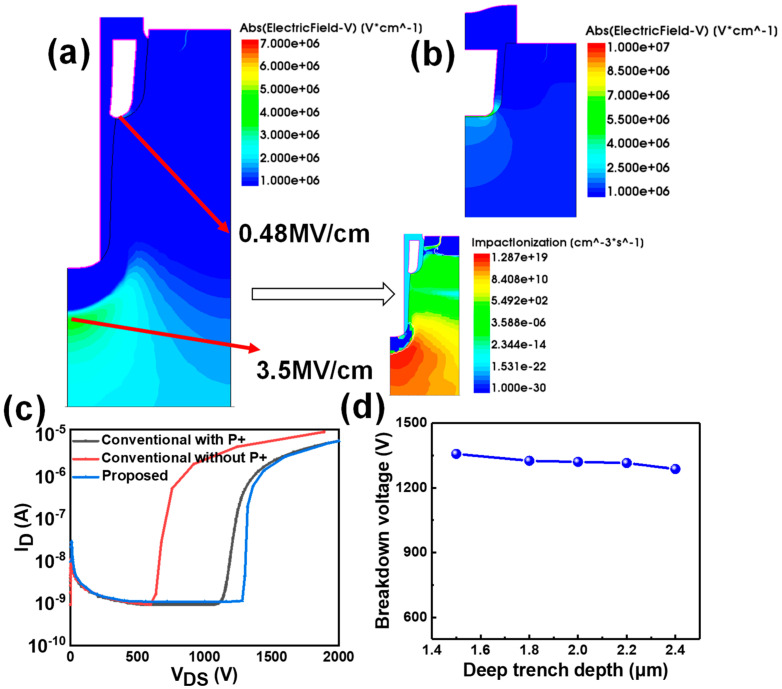
(**a**) The OFF-state electric field distributions in the proposed device at VDS = 1320 V (deep trench depth 2.4 μm). (**b**) The OFF-state electric field distributions in the conventional device without P+ at VDS = 750 V. (**c**) Breakdown characteristic of the proposed device and conventional devices with and without P+, respectively. (**d**) Relationship between deep trench depth and BV of the proposed device.

Figure 3a displays the electric field distributions in the OFF-state (VDS = 1340 V) for the investigated SiC MOSFETs. It is evident that the high-electric-field region primarily resides within the inverted PN junction formed by the P+ region and the drift region, as the grounded P+ shield effectively diverts the electric field. The electric field at the trench gate corner has a considerably lower value of 0.41 MV/cm, which is well below the acceptable threshold of 3 MV/cm. In contrast, the electric field in the P+ shield region reaches 3.5 MV/cm, which is approximately nine times greater than that at the gate corner, with an ionization rate of about 1.28 × 10^19^ cm^−3^s^−1^. Consequently, the proposed device effectively suppresses early breakdown at the corner and resolves the degradation of dynamic on-resistance (R_on_) associated with the floating P+ shield. In comparison to the conventional device without the P+ shield, the maximum electric field in the gate oxide of the proposed device decreases by 65.7% (from 3.5 to 1.2 MV/cm), as illustrated in Figure 3b, thereby achieving superior gate oxide reliability. The OFF-state breakdown characteristics of the studied devices are compared in Figure 3c. The BVs of the proposed device and the conventional device with the P+ shield region are above 1.3 kV, while the BV of the device without the P+ shield is only 750 V. The impact of the deep trench on the breakdown characteristics is also investigated in Figure 3d. A slight decrease in the effective thickness of the N-drift region from 1.5 to 2.4 μm results in a minor degradation in the BV. However, this slight decrease in the BV is well justified by the reduction in the gate oxide field, which is the limiting factor for device reliability.

B. Conduction characteristics

**Figure 4:**
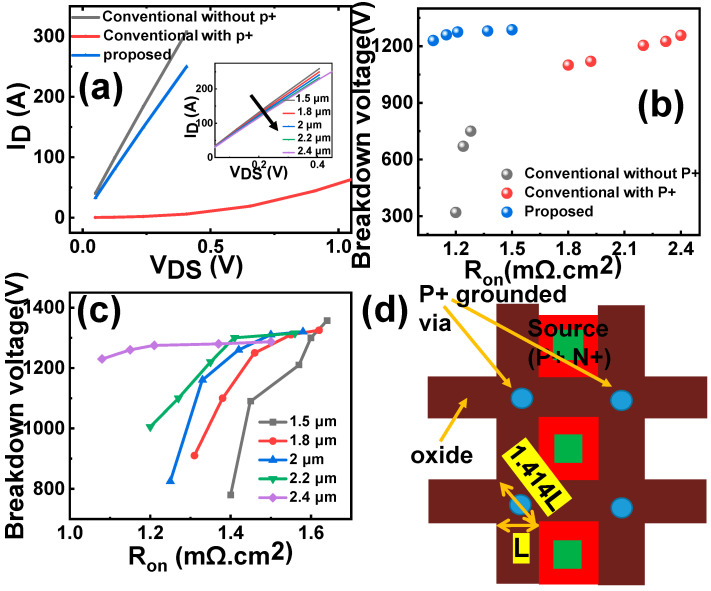
(**a**) ON-state characteristics of the proposed device and conventional devices with and without P+, respectively. (**b**) BV–R_on_ trade-off relationship of the proposed device and conventional devices with and without P+. (**c**) Trade-off relationship between BV and R_on_ with different trench depth. (**d**) Cell layout design of distributed grounding method.

As depicted in Figure 4a, the estimated R_on_ values for the proposed device, conventional device with the P+ shield, and conventional device without the P+ shield are 1.51, 100, and 1.28 mΩ·cm^2^, respectively. The introduction of the P+ shield significantly affects the conduction characteristics by increasing the electron flow resistance near the sidewall. However, the proposed device successfully concentrates the distribution of the P+ region, minimizing the impact on the R_on_. By optimizing the doping profile of the body region, the 17.8% increase in the R_on_ can be offset and improved. The depth of the deep trench has little effect on the R_on_, as illustrated in the inserted picture in Figure 4a. Figure 4b presents a comparison of the trade-off relationship between the BV and R_on_ for the three devices. The proposed device achieves the optimal combination of a high BV and low R_on_ simultaneously. Moreover, a deeper depth allows the inverted PN junction in the OFF state to penetrate further into the drift zone. This enables the possibility of further reducing R_on_ by increasing the doping concentration of the JFET region during epitaxial growth (or by ion implantation), as depicted in Figure 4c, without compromising other characteristics. It should be noted that introducing a grounded deep trench in the device structure will increase the width of the trench, which may impede further reductions in the cell size. To address this challenge, a corresponding layout arrangement is proposed, as depicted in Figure 4d. The proposed layout design distributes grounding at intersections and centers, allowing sufficient space for grounding vias. In this design, the width of the cross-region is set to 1.414 times the length (1.414 L), providing ample room for the incorporation of grounding vias.

By implementing the proposed layout arrangement as described, the device cell pitch can be effectively reduced to 1.8 μm. Furthermore, this reduction in cell pitch is achieved while maintaining a desirable on-resistance (R_on_) value below 0.94 mΩ·cm^2^. This approach allows for the efficient utilization of space, optimizing the device’s overall performance characteristics, and enabling the realization of a compact and high-performing device design.

C. Switching characteristics

**Figure 5:**
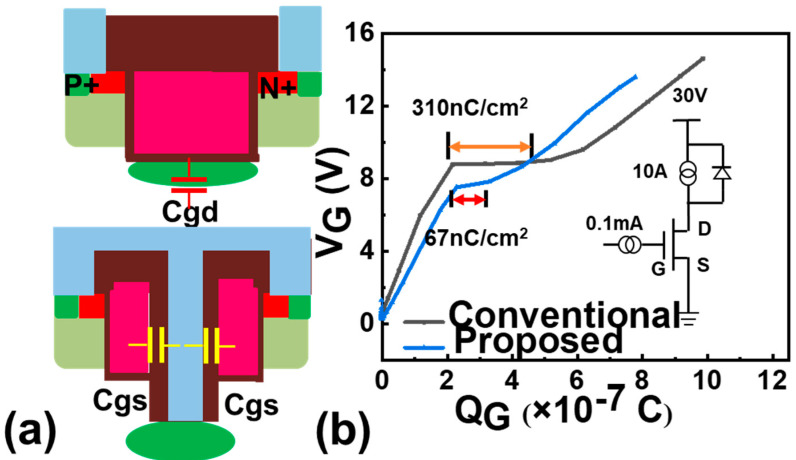
(**a**) Capacitance distribution diagram; (**b**) gate charge of the proposed device and conventional devices with and without P+. The inserted picture shows the test circuit configuration.

The capacitance distribution of the proposed device is shown in Figure 5a. The dominating part of the Miller C_gd_ is decomposed into C_gs_. The results show that the Q_gd_ values for the conventional and the proposed device are 330 nC/cm^2^ and 67 nC/cm^2^, respectively. The gate charge curve of the proposed device has almost no Miller platform. And the increased Q_gs_ can be further reduced by increasing the isolation oxide thickness of the deep trench. Compared with [26], the principle of our device to eliminate Q_gd_ is mainly to convert C_gc_ into C_gs_ through the split gate structure, and the optimization should be better, theoretically. While the reduced Q_G_ improves the switching speed, faster transitions may exacerbate transient oscillations in the gate or drain-source loop due to parasitic inductances. Techniques such as active gate shaping or PT-symmetric damping could be explored to mitigate such effects in future designs [27].

Table 2 summarizes the performance of the proposed and conventional devices. The proposed structure shows an overall better performance including a low R_on_, low Q_gd_, high BV, and high figure of merit FoM (FoM = BV^2^/R_on_).

## 4. Conclusions

In summary, we have introduced a novel SiC MOSFET with a distributed grounded P+ shield. This device features a stepped trench and deep trench server as a P+ shield grounded channel. This way, the inverted p-n junction in the OFF state can be pushed down into the drift region to effectively protect the gate oxide, providing enough margin for optimal doping in the body region. Compared with the conventional P+ shield device, a 78% reduction in the Q_gd_ and 108% increase in the FoM are realized simultaneously. Furthermore, the use of P-type polysilicon in the middle deep trench based on this structure can significantly reduce the Schottky barrier and solve the problem of the insufficient reverse conduction ability of the current body Schottky barrier diode. Overall, this is a technology with practical application prospects. This technology represents a fundamental advancement in SiC power device design by simultaneously solving three longstanding challenges, representing a significant advancement in high-voltage power device technology.

## Figures and Tables

**Table 1:** Key device parameters.

Parameter	Proposed	Conventional
Gate oxide thickness (nm)	50	50
Trench gate depth D1 (μm)	1.0	1.0
D2 (μm)	1.2	
N-JFET doping (cm^−3^)	$4\times 10^{16}$	$4\times 10^{16}$
N-drift doping (cm^−3^)	$7\times 10^{15}$	$7\times 10^{15}$
N-drift thickness (μm)	10	10
Cell pitch L1 (μm)	2.4	2.4
Trench gate width L2 (μm)	1.6	1.6
L3 (μm)	0.3	

**Table 2:** Comparison of performance parameters.

	Proposed	Con with +P	Con Without +P
R_on_ (mΩ·cm^2^)	1.08	1.8	1.28
BV (V)	1230	1100	750
FoM (MW·cm^2^)	1.4	0.672	0.43
Q_gd_ (nC/cm^2^)	67	330	336

## Data Availability

The original contributions presented in this study are included in the article. Further inquiries can be directed to the corresponding author.
